# Supplementary material for: The mechanisms underlying the enhanced high-temperature properties of GRX-810
Source: Nat Commun. 2025 Dec 14;17:963. doi: 10.1038/s41467-025-67687-4 (PMC12847880; doi:10.1038/s41467-025-67687-4)
Supplement: Supplementary file 1 — Supplementary Information [file 41467_2025_67687_MOESM1_ESM.pdf]

Supplementary Information:

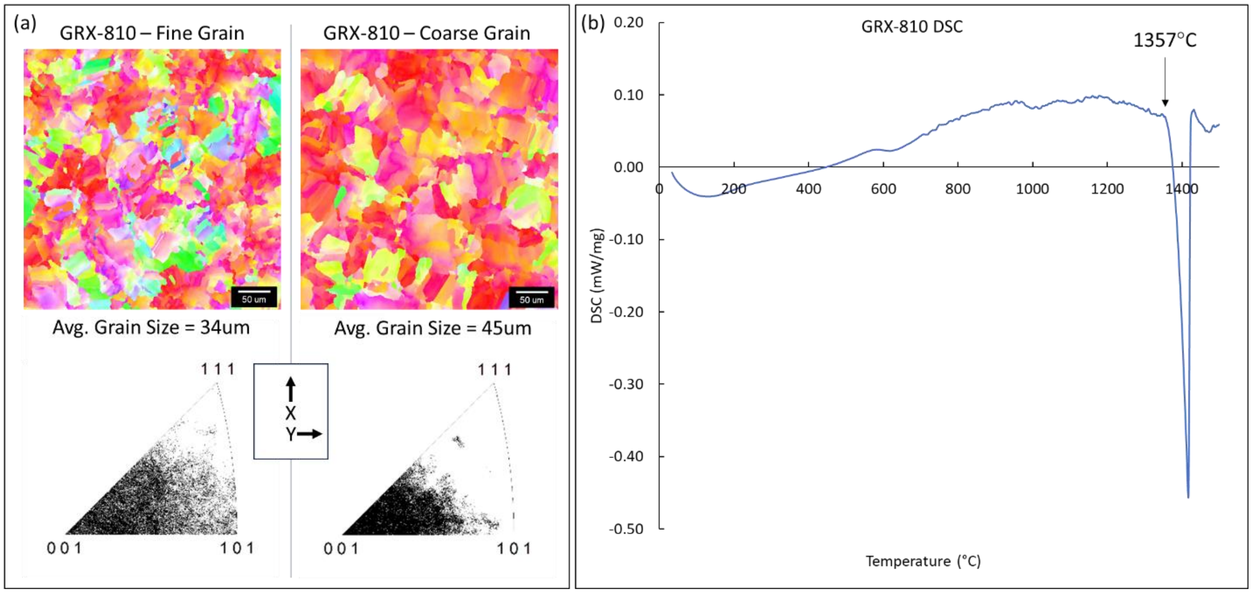

Supplementary Figure 1: Microstructural Analysis and comparison of GRX-810. (a) Electron backscatter diffraction grain orientation maps of as-built Lot 1 GRX-810 produced using an EOS M100 and GRX-810 produced using an EOS M280. The top maps correspond to the XY plane and the bottom maps represent the IPF maps from the orientation map above them. (b) Differential scanning calorimetry (DSC) of FG GRX-810 revealing a solidus of 1357°C

Supplementary Table 1: Tensile Properties of GRX-810. Elevated Tensile data for CG GRX-810. All tests were performed with a 0.305 mm/mm/min strain rate until 4% strain was reached. At 4% elongation the test was transitioned to displacement control at a rate equal to 1.98 mm/mm/min.

Sample	Temperature (C)	Orientation	0.2% Yield Strength (MPa)	Tensile Strength (MPa)	Elongation (%)
GRX810-AB-H	1093.3	Horizontal	117.2	117.2	10.8
GRX810-AB-H	1093.3	Horizontal	114.5	115.1	16.9
GRX810-AB-H	1093.3	Horizontal	117.2	117.9	10.7
GRX810-AB-H	1093.3	Horizontal	116.5	116.5	14
GRX-810-AB-V	1093.3	Vertical	124.8	124.8	43.8
GRX-810-AB-V	1093.3	Vertical	102.0	113.1	37.8
GRX-810-AB-V	1148.9	Vertical	90.5	90.5	11.8
GRX-810-AB-V	1148.9	Vertical	91.7	91.7	19.7
GRX-810-AB-V	1148.9	Vertical	88.9	88.9	13.1
GRX-810-AB-V	1204.4	Vertical	72.7	72.7	12.1
GRX-810-AB-V	1204.4	Vertical	72.8	72.8	8.3
GRX-810-AB-V	1204.4	Vertical	84.6	84.6	10.3
GRX-810-AB-V	1260.0	Vertical	63.9	63.9	6.0
GRX-810-AB-V	1260.0	Vertical	59.0	59.0	7.8
GRX-810-AB-V	1260.0	Vertical	57.8	66.0	7.0
GRX-810-AB-V	1315.6	Vertical	39.8	45.1	3.2
GRX-810-AB-V	1315.6	Vertical	45.5	54.6	4.1
GRX-810-AB-V	1315.6	Vertical	52.4	57.3	5.8
GRX-810-HIP	1093.3	Vertical	100.1	104	20.6
GRX-810-HIP	1093.3	Vertical	103	103.7	27.2
GRX-810-HIP	1093.3	Vertical	102.1	104.9	27.4
GRX-810-HIP	1148.9	Vertical	85.8	91.4	19.6
GRX-810-HIP	1148.9	Vertical	86.5	87.1	33
GRX-810-HIP	1148.9	Vertical	93.1	97.1	24.8
GRX-810-HIP	1204.4	Vertical	69.3	74	14
GRX-810-HIP	1204.4	Vertical	75.8	78.4	16
GRX-810-HIP	1204.4	Vertical	66.2	75.5	21
GRX-810-HIP	1260	Vertical	60	65.5	12.8
GRX-810-HIP	1260	Vertical	55.9	65.6	15.6
GRX-810-HIP	1260	Vertical	62.5	67.2	15
GRX-810-HIP	1315.6	Vertical	32.2	45.5	5.2
GRX-810-HIP	1315.6	Vertical	44.6	48.2	6.6
GRX-810-HIP	1315.6	Vertical	36.5	43.8	4.8

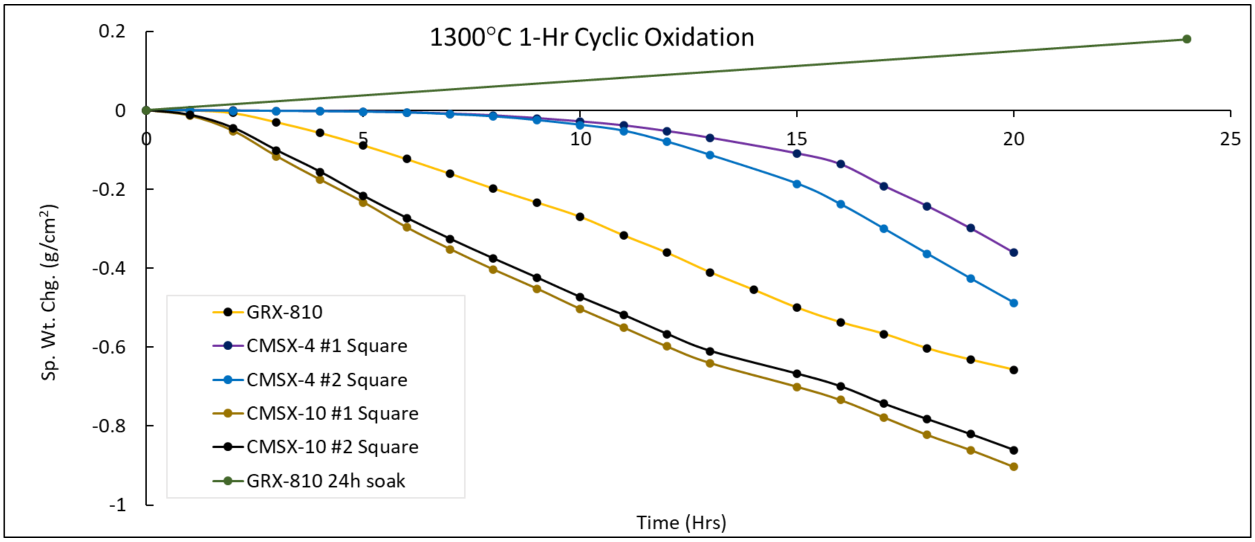

Supplementary Figure 2: Oxidation properties of GRX-810 and single crystal cast superalloys. Additional oxidation results comparing GRX-810 to commonly used single crystal blade alloys at 1300°C**.** There is an additional GRX-810 test showing the weight change after a 24-hour soak at 1300°C.

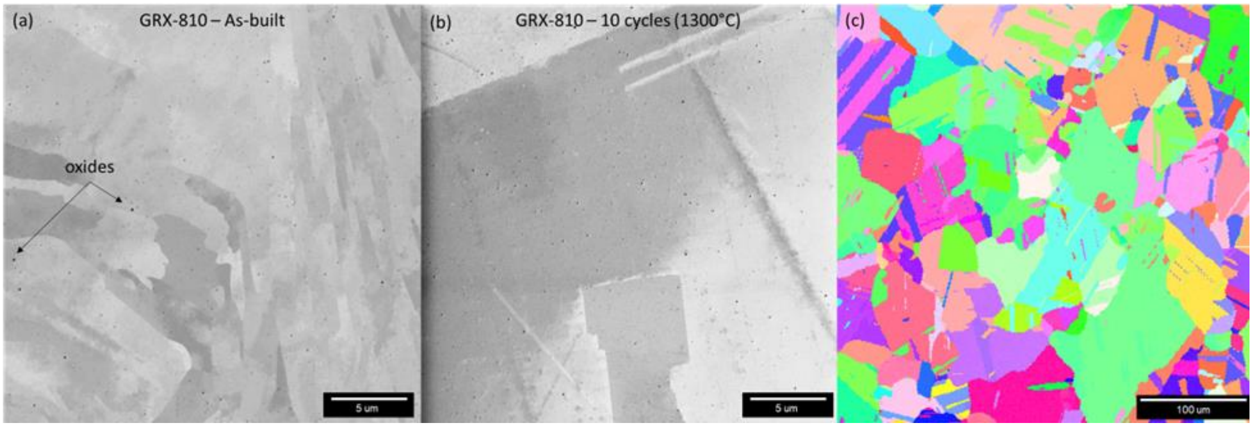

Supplementary Figure 3: Microstrcuture evolution of thermally cycled GRX-810 at 1300°C. SEM micrographs of the oxide (fine dark curcular features) size and morphology of (a) as-printed GRX-810 and (b) GRX-810 after ten 1300°C 1-hour cycles. Grain orientation map of GRX-810 after ten 1300°C 1-hour cycles revealing a recrystallized grain structure.

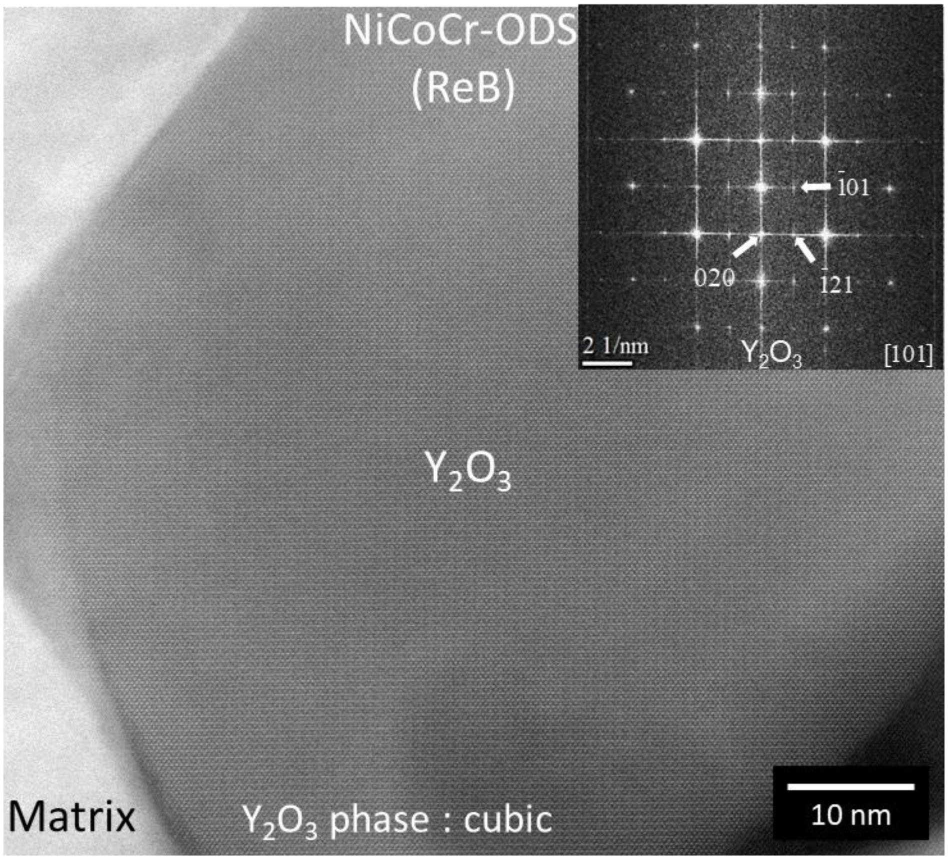

Supplementary Figure 4: High resolution characterization of Yttria particles. Atomic-scale STEM micrograph of the cubis oxides observed in NiCoCr-ReB ODS alloys.

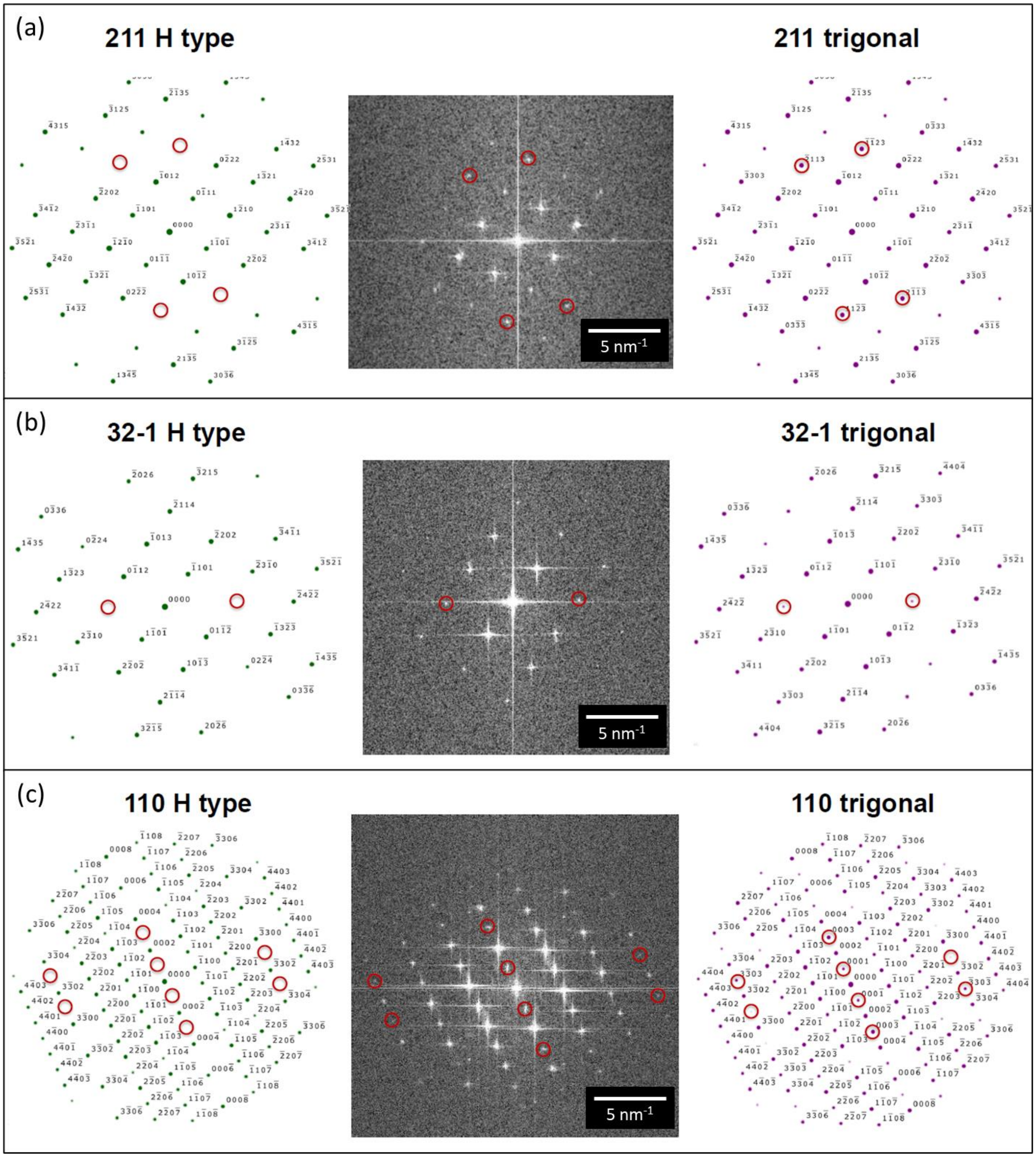

Supplementary Figure 5: GRX-810 yttria crystal structure analysis. The fast fourier transformation of the oxides in GRX-810 at different zone axis compared to both the H (hexagonal) and A type (trigonal) crystal structures of a Y_2O_3 particle.

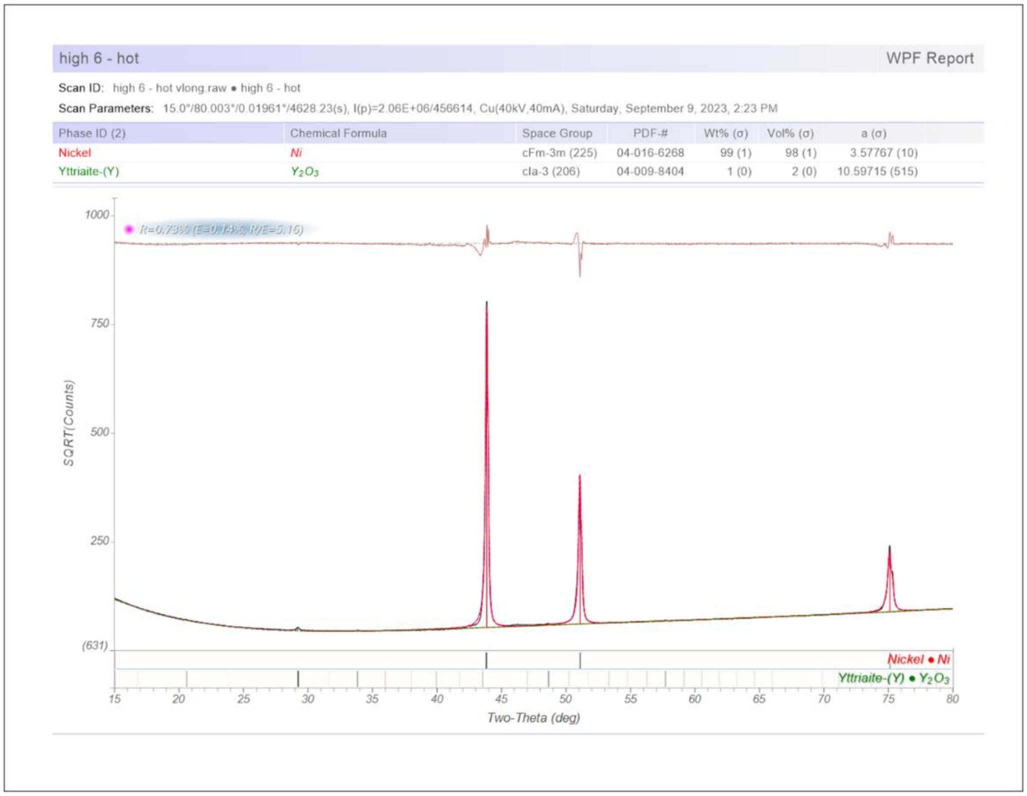

Supplementary Figure 6: XRD of Y_2O_3 coated feedstock. XRD analysis of coated GRX-810 powder.

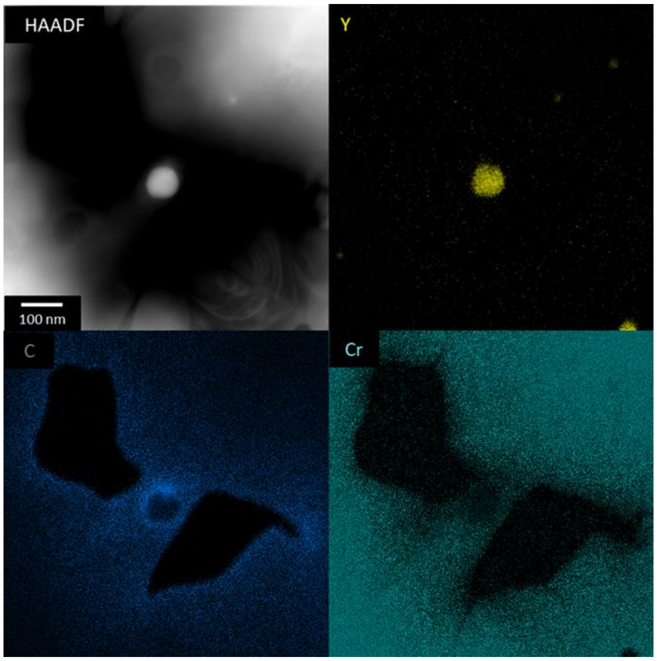

Supplementary Figure 7: Chemical map of oxide/matrix interface. STEM micrograph of an individual oxide particle in GRX-810 and the corresponding Cr, Y, and C chemical maps of the same region. The chemical maps reveal C segregation at the oxide/matrix interface.

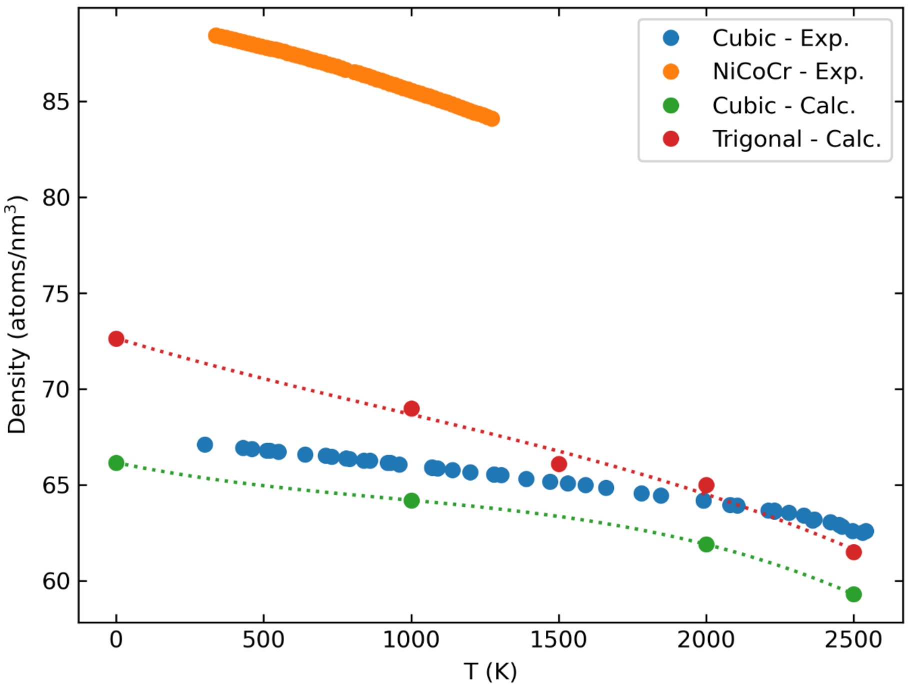

Supplementary Figure 8. **Comparison of Yttria densities.** Density of cubic and trigonal yttria compared with NiCoCr. Experimental data for cubic yttria and NiCoCr are taken from[3] and [4] respectively.

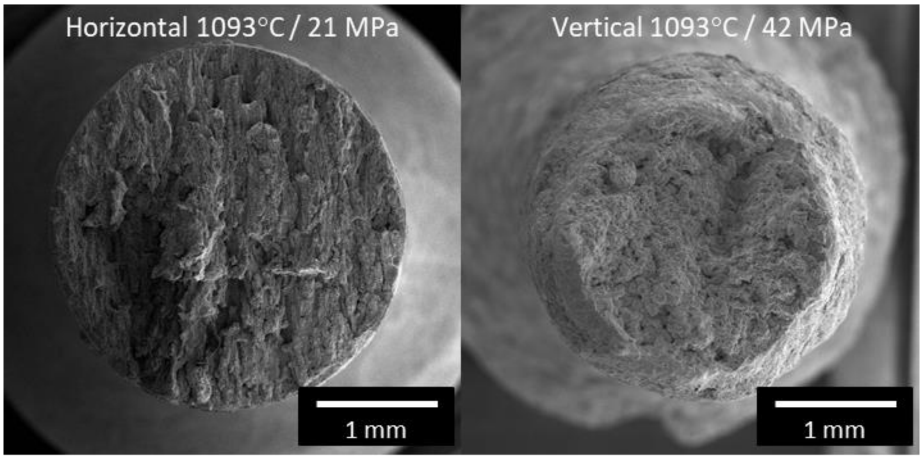

Supplementary Figure 9: Fracture Surface Analysis. Optical microscopy images of creep fracture surfaces in GRX-810 between creep performed in the horizontal and vertical orientations.

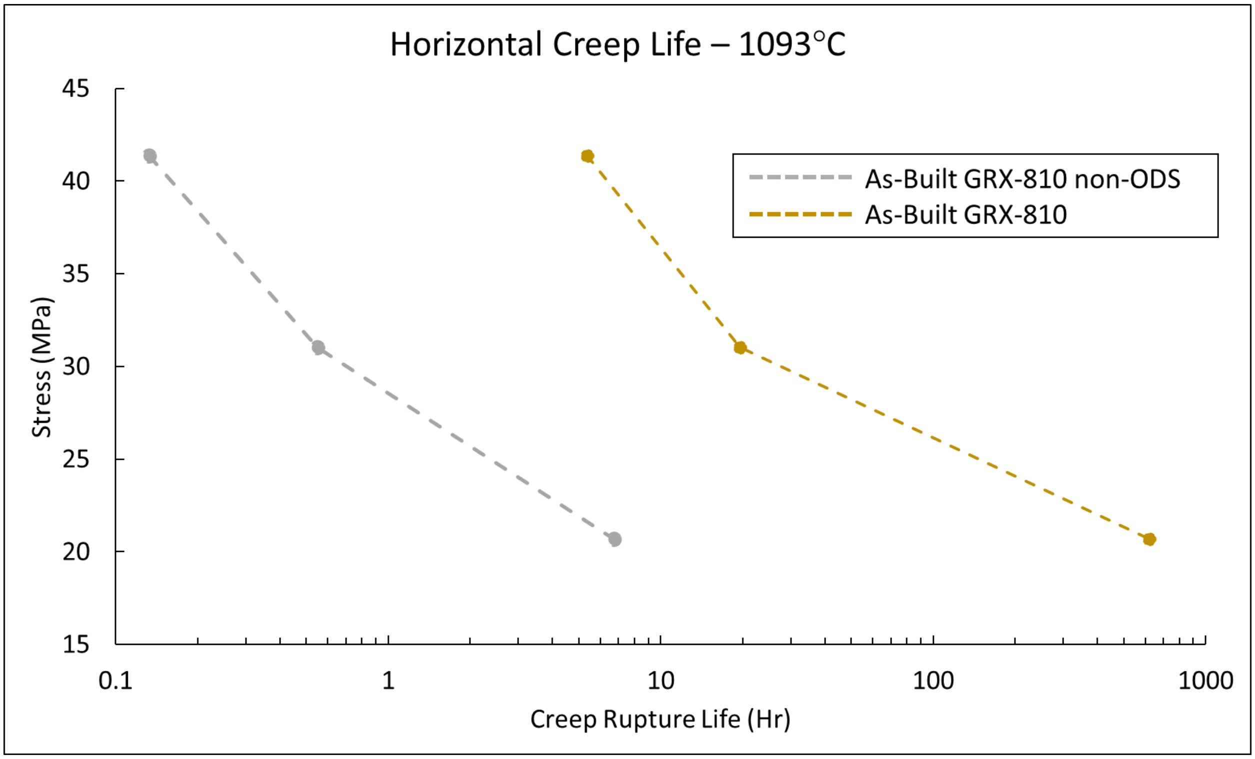

Supplementary Figure 10: Creep life comparison. As-built horizontal creep rupture lives vs stress for GRX-810 with and without oxides.

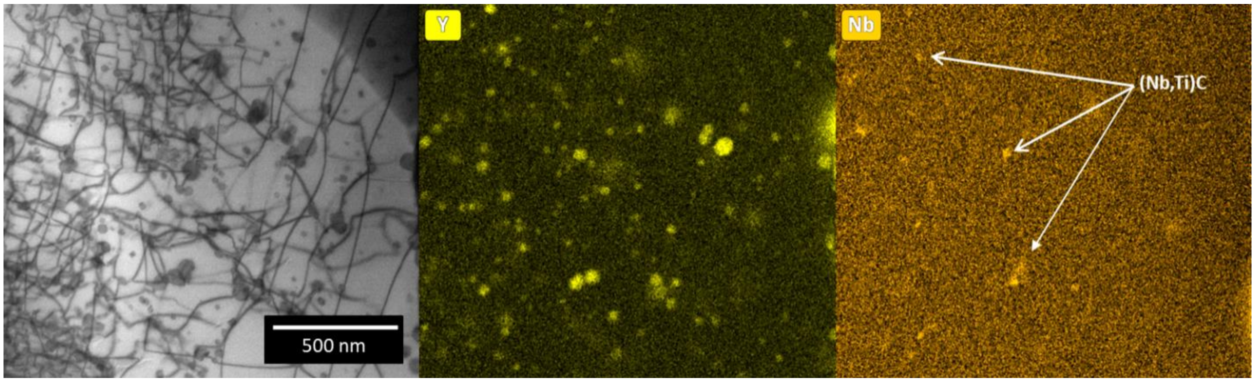

Supplementary Figure 11: **Characterization of oxide and carbide dispersion in post-creep GRX-810.** HAADF STEM image and corresponding Y and Nb maps of a post-creep GRX-810 sample revealing an extensive dislocation network interacting with the nano-oxides and nano-carbides.

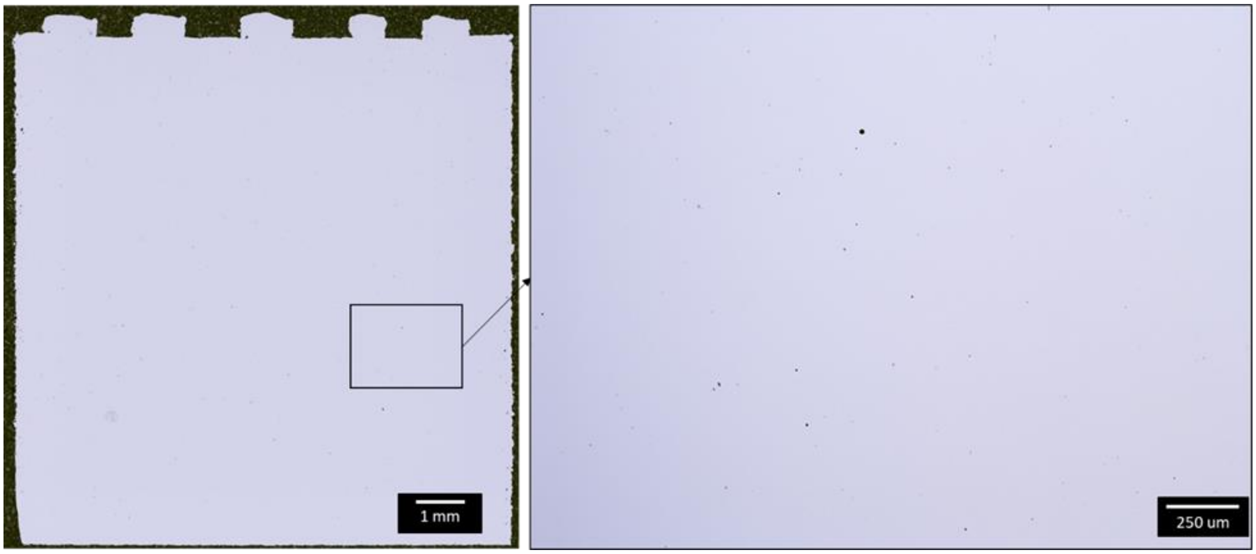

Supplementary Figure 12: **Density of GRX-810**. Optical cross-section of GRX-810 printed on an EOS M280 with a >99.9% density in the as-built state.

Supplementary Table 2: **Superalloy composition(s).** A table of each alloy and its corresponding composition (wt.%) that was referenced and described in the manuscript.

Alloy	Ni	Cr	Co	Mo	W	Nb	Ta	Al	Ti	Re	Fe	B	C	Hf
**Hastelloy X**	41.9	21	1	9	1	0	0	7	1	0	18	0	0.1	0
**Inconel 718**	52.56	19	1	3	0	5	0	0.5	0.9	0	18	0	0.04	0
**Inconel 617**	55.13	22	12.5	9	0	0	0	1	0.3	0	0	0	0.07	0
**Haynes 230**	61.6	22	0	2	14	0	0	0.3	0	0	0	0	0.1	0
**Haynes 188**	22	22	42	0	14	0	0	0	0	0	0	0	0	0
**Inconel 625**	62.95	21.5	0	9	0	3.6	0	0.2	0.2	0	2.5	0	0.05	0
**Haynes 233**	48	19	19	7.5	0.3	0	0.5	3.3	0.5	0	0	0.004	0.1	0
**NiCoCr-ODS**	33.7	30.7	34.7	0	0	0	0	0	0	0	0	0	0	0
**NiCoCr**	34	31	35	0	0	0	0	0	0	0	0	0	0	0
**NiCoCr-ODS (ReB)**	33.0	30.0	34.0	0	0	0	0	0	0	1.5	0	0.003	0	0
**GRX-810 FG**	BAL	30.0	32.1	0	3	0.8	0	0.22	0.30	1.5	0	0	0.05	0
**GRX-810 CG**	BAL	30.6	31.4	0	3.1	0.8	0	0.28	0.29	1.5	0	0	0.05	0
**SC-180**	60.2	5	10	2	5	0	8.5	5.2	1.0	3	0	0	0	0.1
**B-1900+Hf**	63.64	8	10	6	0	0	4.0	6.0	1.0	0	0	0.015	0.1	1.25
**ME3**	50.1	13	20.6	3.8	2.1	0.9	2.4	3.5	3.7	0	0	0.03	0.04	0
**CMSX-4**	BAL	6.5	9.6	0.6	6.4	0	6.5	5.6	1.0	3	0	0	0	0.1
**CMSX-10**	BAL	2.0	3.0	0.4	5.0	0.1	8.0	5.7	0.2	6	0	0	0	0.03

Supplementary Table 3: **Lattice parameters for Yttrium oxides**. Experimental and calculated structural parameters for the cubic and monoclinic phases of Y_2O_3. The cubic lattice parameter is a_0; all others are for the monoclinic phase.

	Experiment (300 K) [8, 9]	Calculated (0 K)
a_0 (Å)	10.604	10.654
a (Å)	13.871	14.051
b (Å)	3.449	3.502
c (Å)	8.586	8.656
β	100.12°	100.41°
$E_{mono} - E_{cubic}$ (meV/atom)		48.8

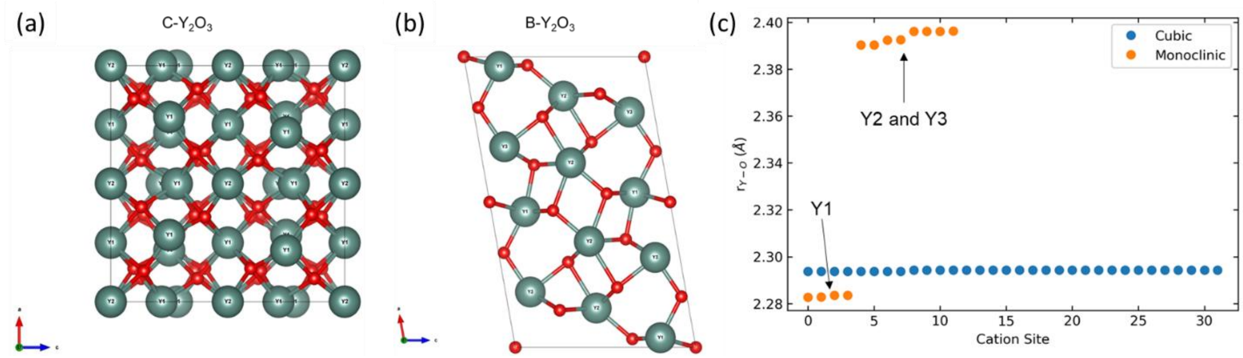

Supplementary Figure 13: **Relaxed structures for DFT calculations**. Relaxed unit cells of cubic (a) and monoclinic (b) Y_2O_3 with the different cationic sites labeled.
