# Supplementary material for: The mechanisms underlying the enhanced high-temperature properties of GRX-810
Source: Nat Commun. 2025 Dec 14;17:963. doi: 10.1038/s41467-025-67687-4 (PMC12847880; doi:10.1038/s41467-025-67687-4)
Supplement: Supplementary file 2 — Description of Additional Supplementary Files [file 41467_2025_67687_MOESM2_ESM.pdf]

Description of Additional Supplementary Files

File Name: Supplementary Data 1
Description: DFT data sets
